# Modulation of Wnt5a Expression by Periodontopathic Bacteria

**DOI:** 10.1371/journal.pone.0034434

**Published:** 2012-04-02

**Authors:** Hiromi Nanbara, Nawarat Wara-aswapati, Toshiyuki Nagasawa, Yasuhiro Yoshida, Reiko Yashiro, Yukiko Bando, Hiroaki Kobayashi, Janjura Khongcharoensuk, Doosadee Hormdee, Waranuch Pitiphat, Jason A. Boch, Yuichi Izumi

**Affiliations:** 1 Department of Periodontology, Tokyo Medical and Dental University, Tokyo, Japan; 2 Global Center of Excellence Program, International Research Center for Molecular Science in Tooth and Bone Diseases, Tokyo Medical and Dental University, Tokyo, Japan; 3 Department of Periodontology, Faculty of Dentistry, Khon Kaen University, Khon Kaen, Thailand; 4 Department of Immunology, School of Medicine, University of Occupational and Environmental Health, Fukuoka, Japan; 5 Department of Community Dentistry, Faculty of Dentistry, Khon Kaen University, Khon Kaen, Thailand; 6 Department of Oral Medicine, Infection and Immunity, Harvard School of Dental Medicine, Boston, Massachusetts, United States of America; East Carolina University School of Medicine, United States of America

## Abstract

Wingless proteins, termed Wnt, are involved in embryonic development, blood cell differentiation, and tumorigenesis. In mammalian hematopoiesis, Wnt signaling is essential for stem-cell homeostasis and lymphocyte differentiation. Recent studies have suggested that these molecules are associated with cardiovascular diseases, rheumatoid arthritis, and osteoarthritis. Furthermore, Wnt5a signaling is essential for the general inflammatory response of human macrophages. Periodontitis is a chronic inflammatory disease caused by gram-negative periodontopathic bacteria and the resultant host immune response. Periodontitis is characterized by loss of tooth-supporting structures and alveolar bone resorption. There have been no previous reports on Wnt5a expression in periodontitis tissue, and only few study reported the molecular mechanisms of Wnt5a expression in LPS-stimulated monocytic cells. Using RT-PCR, we demonstrated that Wnt5a mRNA expression was up-regulated in chronic periodontitis tissue as compared to healthy control tissue. *P. gingivalis* LPS induced Wnt5a mRNA in the human monocytic cell line THP-1 with a peak at 4 hrs after stimulation. *P. gingivalis* LPS induced higher up-regulation of Wnt5a mRNA than *E. coli* LPS. The LPS receptors TLR2 and TLR4 were equally expressed on the surface of THP-1 cells. *P. gingivalis* LPS induced IκBα degradation and was able to increase the NF-κB binding activity to DNA. *P. gingivalis* LPS-induced Wnt5a expression was inhibited by NF-κB inhibitors, suggesting NF-κB involvement. Furthermore, IFN-γ synergistically enhanced the *P. gingivalis* LPS-induced production of Wnt5a. Pharmacological investigation and siRNA experiments showed that STAT1 was important for *P. gingivalis* LPS-induced Wnt5a expression. These results suggest that the modulation of Wnt5a expression by *P. gingivalis* may play an important role in the periodontal inflammatory process and serve a target for the development of new therapies.

## Introduction

Wingless, a second chromosome recessive mutation in *Drosophila*, was first reported by Sharma and Chopra in 1975 [1]. It was characterized as a segment polarity gene in *Drosophila*, which is essential for embryonic segmentation and patterning [2]. Various homologs of the Wingless protein, termed Wnt, are involved in embryonic development, blood cell differentiation during mammalian hematopoiesis, and tumorigenesis [3]. In mammalian hematopoiesis, Wnt signaling is essential for stem-cell homeostasis [4] and lymphocyte differentiation [5], [6].

Of particular interest is Wnt5a, which was first identified in the mouse [7]. It is secreted by activated antigen-presenting cells and by inflammatory synoviocytes from rheumatoid arthritis joints [8] and atherosclerotic lesions [9]. Wnt5a expression is induced by mycobacterial cell wall components and endotoxin in human antigen-presenting cells [10], and Wnt5a signaling is essential for the general inflammatory response of human macrophages during sepsis [11].

Wnt5a expression is dependent on Toll-like receptor (TLR) signaling and activation of the central inflammatory regulator NF-κB in human antigen-presenting cells [10]. Furthermore, interleukin (IL)-6 family members activate the gp130-JAK-signal transducer and activator of transcription 3 (STAT3) signaling cascade to up-regulate Wnt5a transcription in chronic persistent inflammation and rheumatoid arthritis [12]. However, little is known about the expression and modulation of Wnt homologs in inflammatory settings, such as periodontitis.

Periodontal diseases are infections caused by gram negative periodontopathic bacteria [13] which colonize on the tooth surface at or below the gingival margin. In severe cases, these are accompanied by inflammation of the gingiva and destruction of periodontal tissues, leading to alveolar bone loss. Periodontopathic bacteria produce many virulence factors, such as lipopolysaccharide (LPS) and peptidoglycan those induce host immune responses, including the production of inflammatory cytokines. High levels of several inflammatory cytokines, such as IL-1α, IL-1β, IL-6, interferon-γ (IFN-γ), and tumor necrosis factor α (TNF-α), have been found in the tissue and gingival crevicular fluid of patients with advanced periodontitis [14], [15]. These inflammatory cytokines are reported to stimulate the production of receptor activator for nuclear factor κB ligand (RANKL) [16], [17], which binds to receptor activator for nuclear factor κB (RANK) on the surface of preosteoclasts. This binding stimulates both the differentiation of osteoclast progenitors and the activity of mature osteoclasts leading to alveolar bone resorption [18]–[21].

The purpose of this study was to evaluate the levels of Wnt5a expression in chronic periodontitis tissue and investigate the modulation of Wnt5a expression by periodontopathic bacteria. We show that Wnt5a mRNA was significantly up-regulated in chronic periodontitis tissue when compared to non-periodontitis tissue. The expression of Wnt5a was dependent on the NF-κB pathway and was partly mediated by the STAT1 signaling pathway in response to *P. gingivalis* LPS/IFN-γ in the human monocytic cell line THP-1. This result suggests that the modulation of Wnt5a expression by *P. gingivalis* may play an important role in the periodontal inflammatory process.

## Results

### Wnt5a was significantly up-regulated in chronic periodontitis tissues

**Figure 1:**
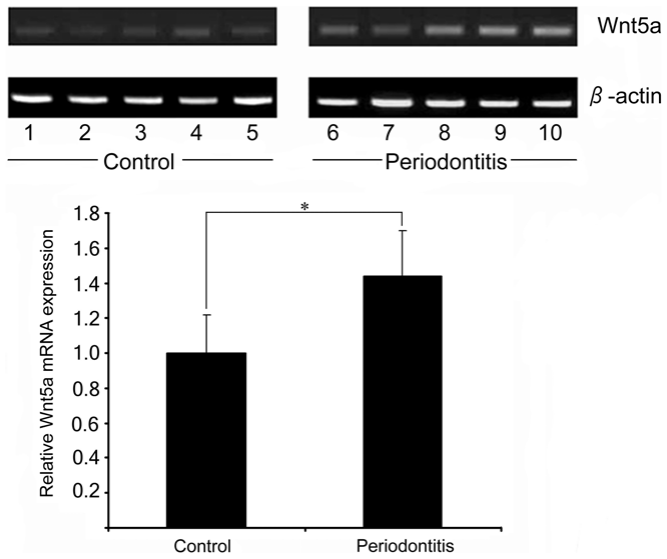
The levels of Wnt5a mRNA were significantly up-regulated in chronic periodontitis tissues. Upper panel; Total RNA was extracted from periodontitis tissues, and the expression of Wnt5a mRNA was detected by RT-PCR. PCR products were electrophoresed on a 1.5% agarose gel and visualized by UV illumination. β-actin served as the internal control. Results are representative of five patients (right panel). Lower panel; The relative mRNA levels of Wnt5a. The horizontal line within each box represents the median expression level in each group.

Wnt5a signaling is known to be essential for the general inflammatory response [11] and it is secreted in chronic inflamed site such as inflammatory synoviocytes [8], the atherosclerotic lesions [9], and the serum and bone marrow of patients with severe sepsis [11]. Table 1 summarizes the characteristics of the study subjects and sampling sites. Subjects in the periodontitis group were significantly older, and had higher mean PD, mean CAL and proportion of BOP-positive sites as compared with the control group. Production of Wnt5a mRNA was detected in all gingival tissue samples (Fig. 1). The mean relative mRNA level of Wnt5a was significantly higher in the periodontitis group (1.44±0.26) than in the control group (1.00±0.22; p<0.001). Additional regression analysis controlling for the effect of age confirmed these results that chronic periodontitis was associated with increased mRNA levels of Wnt5a (p<0.001).

**Table 1:** Characteristics of the Study Subjects.

Charactereistic	Comparison group		
	Control group	Periodontitis group	p-value*
	(n = 14)	(n = 13)	
Age in years	31.0±13.1	43.5±13.0	0.02
(mean ± SD)			
Female	12 (85.7)	10 (76.9)	0.65
n (%)			
Probing depth**	2.0±0.9	9.5±1.9	<0.001
(mm; mean ± SD)			
Clinical attachment level**	0.8±0.7	10.5±2.6	<0.001
(mm; mean ± SD)			
Bleeding on probing*	3 (21.4)	13 (100)	<0.001
n (%)			

* Mann-Whitney U test for continuous data, and Fisher's exact test for binary data.

** Measured at the sampling sites.

### Wnt5a mRNA was expressed by the human monocytic cell line THP-1 in response to *P. gingivalis* LPS

**Figure 2:**
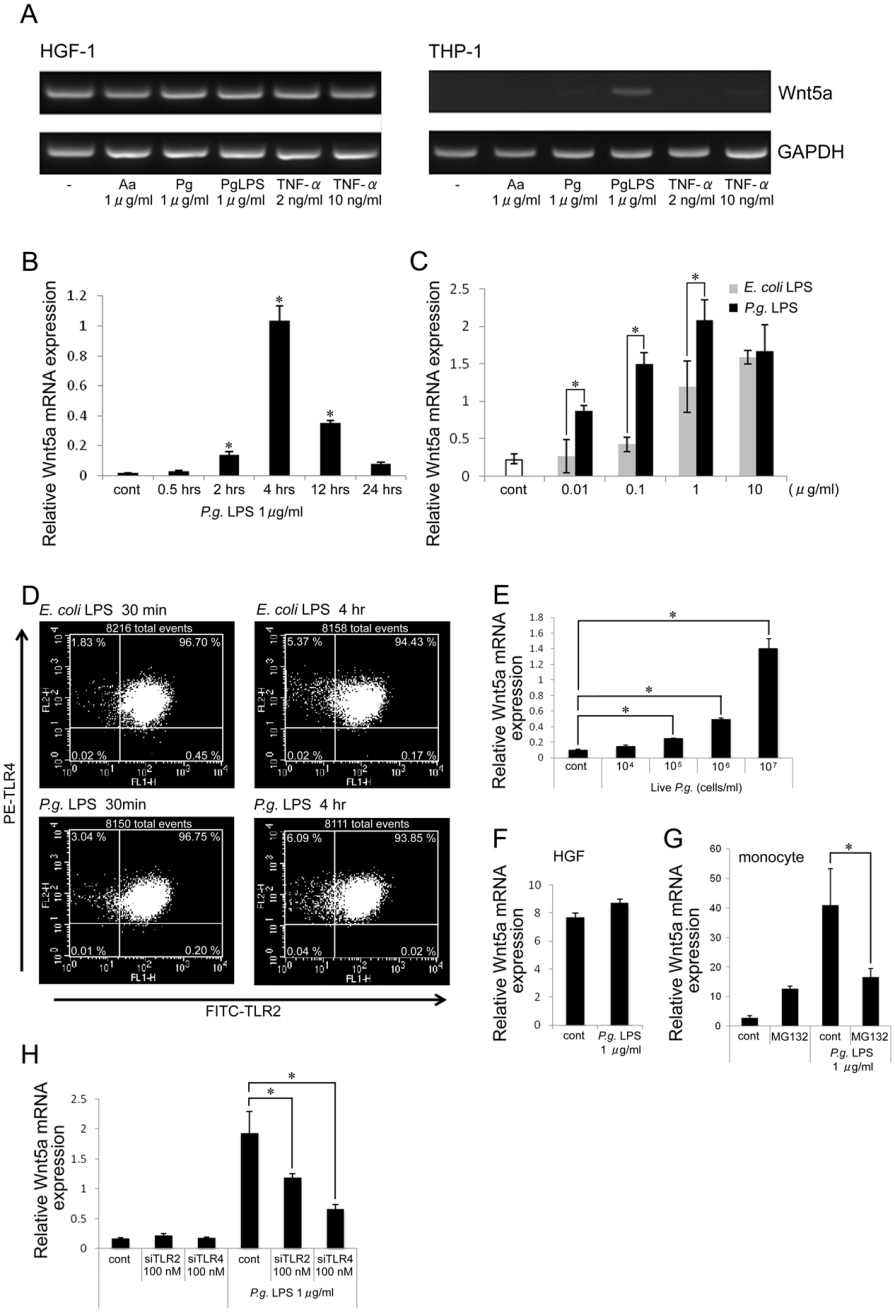
Wnt5a was specifically up-regulated in THP-1 cells by *P. gingivalis* LPS. (A) HGF-1 and THP-1 cells were stimulated with *A. actinomycetemcomitans* sonicated extract, *P. gingivalis* sonicated extract, *P. gingivalis* LPS, and TNF-α for 4 hrs, and the expression of Wnt5a mRNA was detected by RT-PCR. PCR products were electrophoresed on a 1.5% agarose gel and visualized by UV illumination. GAPDH served as the internal control. (B) THP-1 cells were stimulated with 1 µg/ml of *P. gingivalis* LPS for 0.5, 2, 4, 12, or 24 hrs, and the expression of Wnt5a mRNA was detected by real-time PCR. Relative expression levels of Wnt5a are shown. (C) THP-1 cells were stimulated with 0.01–10 µg/ml of *P. gingivalis* LPS (black bars) or *E. coli* LPS (gray bars) for 4 hrs, and then the expression of Wnt5a mRNA was detected by real-time PCR. Relative expression levels of Wnt5a are presented. (D) THP-1 cells were stimulated with *E. coli* 055:B5 LPS (middle and right upper panels) or *P. gingivalis* LPS (middle and right lower panels) for 30 min or 4 hrs, and then the expression of surface TLR2 and TLR4 protein was determined by flow cytometry. Left upper panel shows no-staining condition, and left lower panel shows un-stimulated condition with staining. (E, F, G, H) The expression of Wnt5a mRNA was detected by real-time PCR. Relative expression levels of Wnt5a mRNA are shown. (E) THP-1 cells were stimulated with 10^4^–10^7^ cells/ml of live *P. gingivalis* for 4 hrs. (F, G) Primary human gingival fibroblasts (HGF) and human monocytes were stimulated with 1 µg/ml of *P. gingivalis* LPS for 4 hrs. Monocytes were pretreated with the NF-κB inhibitor MG132 for 1 hr. Here we describe typical dates of three samples. (H) THP-1 cells were stimulated with *P. gingivalis* LPS for 4 hrs after being transfected with TLR2 siRNA, TLR4 siRNA or control siRNA for 72 hrs. *p<0.05.

The human gingival fibroblast cell line HGF-1 and the human monocytic cell line THP-1 were stimulated with *A. actinomycetemcomitans* sonicated extract, *P. gingivalis* sonicated extract, *P. gingivalis* LPS, or TNF-α for 4 hrs. Our results showed that the expression of Wnt5a mRNA in HGF-1 cells was constant in response to different treatments (Fig. 2A). However, in THP-1 cells, Wnt5a mRNA was strongly induced by *P. gingivalis* LPS but was only slightly increased by *P. gingivalis* sonicated extracts or a high concentration of TNF-α. Live *P. gingivalis* also significantly increased the expression of Wnt5a mRNA in THP-1 (Fig. 2E). When THP-1 cells were stimulated with various concentrations of *P. gingivalis* LPS or *E. coli* LPS (Fig. 2C), the maximum Wnt5a mRNA expression occurred after stimulation with 1 µg/ml of *P. gingivalis* LPS. *P. gingivalis* LPS could induce more potent Wnt5a mRNA expression than *E. coli* LPS. When THP-1 cells were stimulated with 1 µg/ml of *P. gingivalis* LPS for 0.5, 2, 4, 12, and 24 hrs, the maximum expression of Wnt5a mRNA occurred at 4 hrs after stimulation (Fig. 2B). Flow cytometry demonstrated that TLR2 and TLR4 were equally expressed on the surface of THP-1 cells stimulated by *P. gingivalis* LPS and *E. coli* LPS, suggesting that the expression of these receptors were not changed by the stimulation (Fig. 2D). *P. gingivalis* LPS-induced Wnt5a mRNA was significantly reduced by either TLR2 siRNA or TLR4 siRNA (Fig. 2H), suggesting that *P. gingivalis* LPS used in this study utilized both TLR2 and TLR4.

In primary human gingival fibroblasts (HGF), the expression of Wnt5a mRNA was rather constant after stimulation with *P. gingivalis* LPS (Fig. 2F). On the contrary, in primary monocytes, Wnt5a mRNA expression was significantly increased by *P. gingivalis* LPS and was reduced by using NF-κB inhibitor MG132 (Fig. 2G). These results suggest that monocytes, but not HGF, play an important role in Wnt5a up-regulation at inflamed site.

### The *P. gingivalis*-LPS induced Wnt5a expression is dependent upon NF-κB signaling

**Figure 3:**
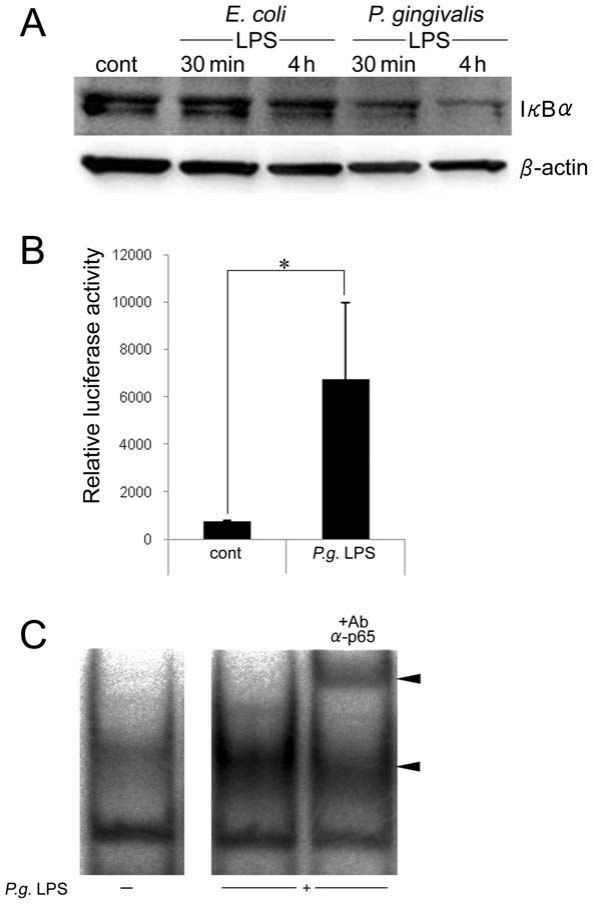
Induction of Wnt5a expression is NF-κB dependent. (A) THP-1 cells were stimulated with *E. coli* LPS or *P. gingivalis* LPS for 30 mins or 4 hrs. Whole cell extracts were prepared and analyzed by Western blot by using antibodies against IκBα. β-actin served as the protein loading control. (B) THP-1 cells were stimulated with *P. gingivalis* LPS for 4 hrs after being transfected with NF-κB reporter plasmid for 12 hrs, and the transcription activity was assessed by luminometer. The activity is represented by the relative luciferase activity. (C) Nuclear extracts were prepared and EMSA was performed with γ^32^P-labeled oligonucleotides representing the NF-κB consensus sequence as a probe. Anti-p65 antibody was used for supershift assays. The lower arrow shows the DNA-protein complex, and the upper arrow shows the supershifted band. *p<0.05.

A recent study showed that Wnt5a expression is dependent on the activation of the central inflammatory regulator NF-κB [10]. We examined the degradation of IκBα in THP-1 cells stimulated with *P. gingivalis* LPS. After 30 mins or 4 hrs, whole cell lysates were subjected to Western blot. In unstimulated THP-1 cells, IκBα was abundant. In contrast, stimulation with *P. gingivalis* LPS markedly reduced the cytosolic level of IκBα (Fig. 3A). To examine *P. gingivalis* LPS-induced NF-κB activity, THP-1 cells were transfected with a luciferase reporter plasmid containing the NF-κB binding site for 18 hrs. After treatment with *P. gingivalis* LPS, the luciferase activity was measured with a luminometer. *P. gingivalis* LPS-induced NF-κB activity was significantly upregulated in THP-1 cells (Fig. 3B). Further, the ability of NF-κB to bind to DNA was analyzed by electrophoretic mobility shift assay (EMSA). The [γ-^32^P]-labeled probe, which contained the NF-κB binding consensus sequence, was incubated with the nuclear extract of THP-1 cells stimulated with *P. gingivalis* LPS for 40 minutes. Protein-DNA complex bands (Fig. 3C, lower arrow) were supershifted by the addition of anti-p65 antibody (Fig. 3C, upper arrow). This result suggests that *P. gingivalis* LPS is able to increase the NF-κB binding activity to DNA.

**Figure 4:**
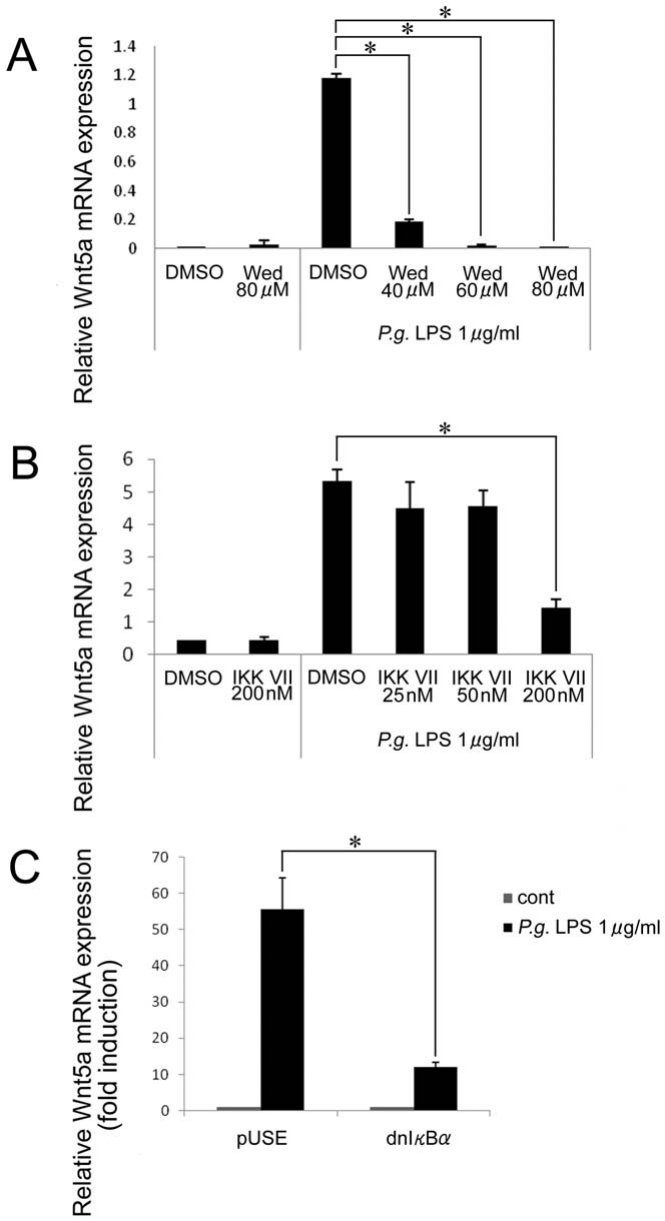
*P. gingivalis* LPS-induced Wnt5a expression was inhibited by wedelolactone or dnIκBα. (A, B) THP-1 cells were pre-treated with the IKK inhibitor wedelolactone or the IKK inhibitor VII for 1 hr. The expression of Wnt5a mRNA was detected by real-time PCR. Relative expression levels of Wnt5a are presented. DMSO is a solvent control for both inhibitors. (C) THP-1 cells were transfected with a dominant-negative form of IκBα (dnIκBα) or the parent plasmid (pUSE) for 12 hrs. Fold induction of Wnt5a mRNA expression is shown. *p<0.05.

The addition of wedelolactone or IKK inhibitor VII, which act as selective irreversible inhibitors of IKKα or IKKβ kinase activities, inhibited *P. gingivalis* LPS-induced Wnt5a mRNA in a dose-dependent manner expression (Fig. 4A, 4B). Moreover, the effect of an expression vector for a dominant-negative form of IκBα was investigated. The expression of *P. gingivalis* LPS-induced Wnt5a mRNA was inhibited by transfection with a dominant-negative IκBα plasmid (Fig. 4C). Taken together, our results indicate that the induction of Wnt5a mRNA expression by *P. gingivalis* LPS is dependent upon NF-κB signaling.

### The induction of Wnt5a is partly mediated via the JAK/STAT signaling pathway

**Figure 5:**
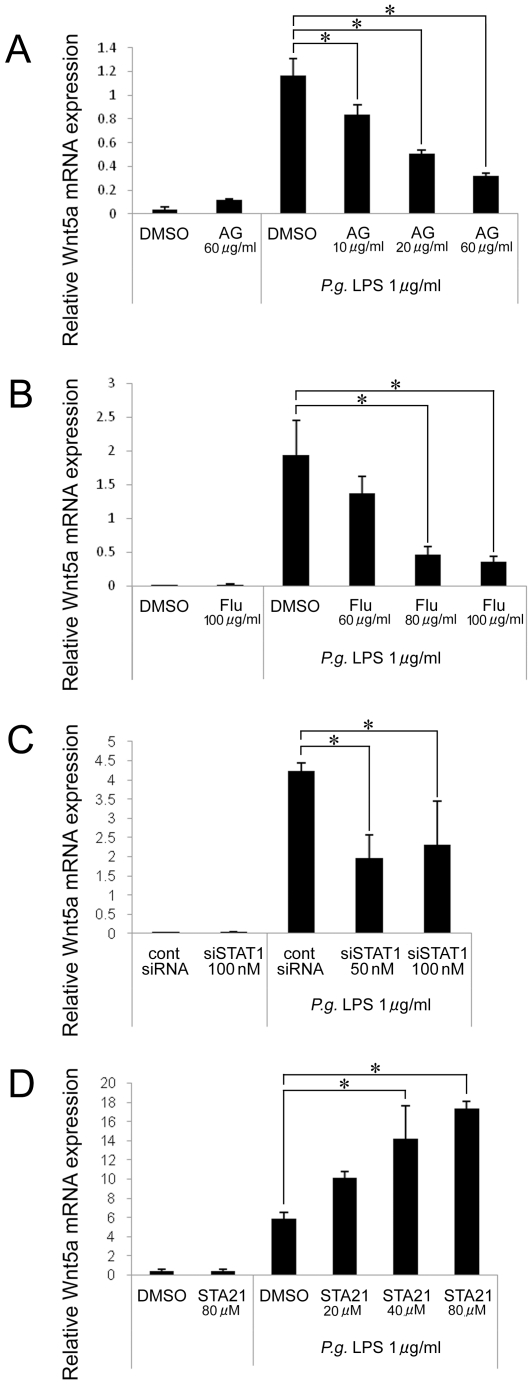
Induction of Wnt5a expression is partly JAK/STAT dependent. (A–D) The expression of Wnt5a mRNA was detected by real-time PCR. Relative expression levels of Wnt5a are presented. (A, B) THP-1 cells were pre-treated with AG490 (A) or fludarabine (B) for 1 hr and then stimulated with *P. gingivalis* LPS for 4 hrs. DMSO is a solvent control for both inhibitors. (C) THP-1 cells were stimulated with *P. gingivalis* LPS for 4 hrs after transfection with STAT1 siRNA or control siRNA for 18 hrs. (D) THP-1 cells were pre-treated with STA21 for 1 hr and stimulated with *P. gingivalis* LPS for 4 hrs. *p<0.05.

Tandem STAT3-binding sites in the *Wnt5a* gene have been identified [22]. We examined the role of the JAK/STAT pathway in the regulation of Wnt5a mRNA expression. AG490 (JAK/STAT inhibitor), fludarabine (STAT1 inhibitor), and STA21 (STAT3 inhibitor) were used to inhibit the JAK/STAT pathway 1 hr prior to *P. gingivalis* LPS stimulation. Pretreatment with either AG490 or fludarabine significantly decreased Wnt5a mRNA expression in THP-1 cells in a dose-dependent manner (Fig. 5A, B). Additionally, STAT1 siRNA was able to suppress the induction of Wnt5a by *P. gingivalis* LPS as compared to control siRNA (Fig. 5C). However, the *P. gingivalis* LPS-induced Wnt5a mRNA expression was augmented by a STAT3 inhibitor, STA21 (Fig. 5D). Our results suggest that STAT1 positively regulated induction of Wnt5a by *P. gingivalis* LPS.

### Wnt5a mRNA expression was increased by co-stimulation with *P. gingivalis* LPS and IFN-γ

**Figure 6:**
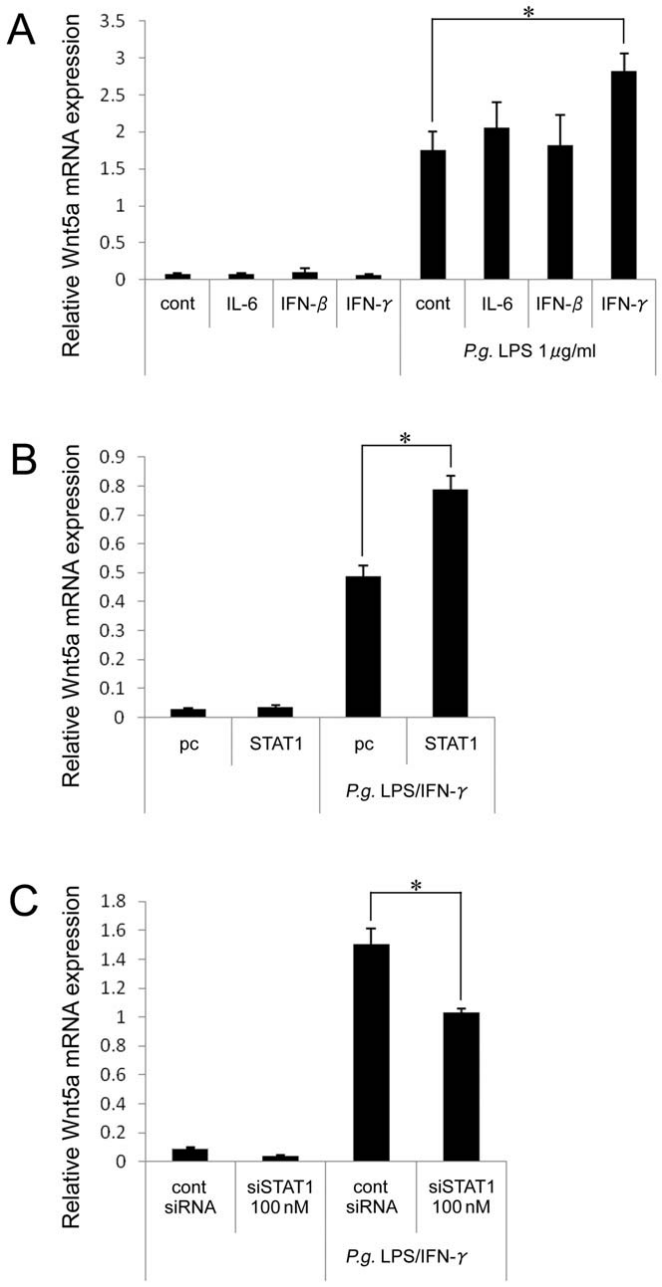
The expression of Wnt5a was increased by co-stimulation with *P. gingivalis* LPS and IFN-γ. (A–C) The expression of Wnt5a mRNA was detected by real-time PCR. Relative expression levels of Wnt5a are presented. (A) THP-1 cells were stimulated with 10 ng/ml of IL-6, 10 ng/ml of IFN-β or 10 ng/ml of IFN-γ with or without *P. gingivalis* LPS for 4 hrs. (B) THP-1 cells were transfected with wild-type STAT1 expression vector or parent plasmid 12 hrs prior to stimulation with *P. gingivalis* LPS/IFN-γ for 4 hrs. (C) THP-1 cells were stimulated with *P. gingivalis* LPS for 4 hrs after being transfected with STAT1 siRNA or control siRNA for 18 hrs. *p<0.05.

To investigate the role of the JAK/STAT pathway in detail, we examined the expression of Wnt5a mRNA after co-stimulation with *P. gingivalis* LPS and either IL-6, IFN-β, or IFN-γ. IL-6, IFN-β, or IFN-γ alone did not elicit the induction of Wnt5a mRNA when compared to the control. Wnt5a mRNA levels were up-regulated in the presence of *P. gingivalis* LPS alone. Furthermore, *P. gingivalis* LPS together with IFN-γ synergistically activated Wnt5a mRNA expression (Fig. 6A).

IFN-γ induces the phosphorylation of STAT1, which forms STAT1 homodimers. The effects of an expression vector for wild-type STAT1 on Wnt5a expression were investigated. After transfection, THP-1 cells were co-stimulated with *P. gingivalis* LPS and IFN-γ. The expression of Wnt5a mRNA was markedly increased by transfection with a wild-type STAT1 expression plasmid (Fig. 6B). Additionally, STAT1 siRNA significantly suppressed the Wnt5a mRNA expression induced by *P. gingivalis* LPS and IFN-γ (Fig. 6C). These results suggest that the induction of Wnt5a mRNA by co-stimulation with *P. gingivalis* LPS and IFN-γ is partly mediated via STAT1 signaling.

## Discussion

In this study, we investigated the levels of Wnt5a expression in chronic periodontitis tissues, and the modulation of Wnt5a expression by periodontopathic bacteria. Wnt5a mRNA was significantly up-regulated in chronic periodontitis tissues as compared to healthy control tissues. Chronic inflammatory periodontitis tissue comprises of epithelial cells, gingival fibroblasts and inflammatory cells including PMNs, monocytes and macrophages. The expression of Wnt5a was dependent upon the NF-κB signaling pathway and was partly mediated by the STAT1 signaling pathway in response to *P. gingivalis* LPS/IFN-γ in the human monocytic cell line THP-1.

Wnt5a is secreted by activated antigen-presenting cells [8], [10], [11] and said to be induced by *M. tuberculosis*, *M. avium*, *S. aureus* or *S. typhimurium* [10], [23]. The expression of Wnt5a mRNA in response to periodontopathic bacterial stimuli was also observed in periodontal tissue cells. Whereas the expression of Wnt5a was constant in response to different treatments in HGF-1 cells, it was specifically up-regulated by *P. gingivalis* LPS and was slightly increased by *P. gingivalis* sonicated extracts in the human monocytic cell line THP-1. Sonicated extract of *P. gingivalis* comprises of various components including protein, lipid, LPS and other factors, which have different properties to stimulate host immune responses. But *P. gingivalis* LPS from InvivoGen is ultra-pure LPS (according to the manufacturer) and thus possesses a high concentration of LPS. In our study, primary cells including monocytes and gingival fibroblasts responded to *P. gingivalis* LPS in a similar manner to the cell lines (THP-1 and HGF-1).

*P. gingivalis* is considered to be an important member of the periodontopathic bacteria involved in periodontal disease [24], [25] leading to inflammation and destruction of alveolar bone and the supporting connective tissue surrounding teeth. *P. gingivalis* is a gram-negative obligate anaerobic rod bacterium that has virulence factors such as LPS [24]. Although TLR4 has been reported to function as an LPS signaling receptor, *P. gingivalis* LPS is less dependent on TLR4 signaling than *Escherichia coli* LPS [26]. Lipid A of *P. gingivalis* differs both structurally and functionally from *E. coli* lipid A [24], and *P. gingivalis* LPS utilizes either TLR2 and TLR4 to mediate inflammatory signaling [27]. Indeed, in this study, the expression of Wnt5a mRNA was down-regulated by TLR2 siRNA and TLR4 siRNA. The engagement of TLR activates a variety of intracellular signaling pathways that ultimately activate the transcriptional factor NF-κB [28]. In this study, the expression of Wnt5a mRNA induced by *P. gingivalis* LPS was higher than the expression induced by *E. coli* LPS. However, the expression of TLR2 and TLR4 receptors on the surface of THP-1 cells was not changed after stimulation with *P. gingivalis* LPS or *E. coli* LPS. These results suggest that the difference in Wnt5a mRNA expression level between *P. gingivalis* LPS and *E. coli* LPS is not dependent upon the expression of TLR. Generally, activated NF-κB induces IκBα de novo, then IκBα will be comparable level to no stimulation as seen in *E. coli* LPS (Fig. 3A). It is not clear but there might be some involvement in strong induction of Wnt5a by *P. gingivalis* LPS in comparison with *E. coli* LPS. In addition, *P. gingivalis* LPS is said to induce lower IL-10 than *E. coli* LPS in dendritic cells generated from human peripheral monocytes [29] suggesting that down-regulation of Wnt5a by *P. gingivalis* LPS-induced IL-10 may be weaker than *E. coli* LPS.

In this study, we found that AG490 and Fludarabine, but not STA21, were capable of suppressing *P. gingivalis* LPS-mediated Wnt5a up-regulation in THP-1 cells. Fludarabine is used in the treatment of various hematological malignancies, particularly B-cell chronic lymphocytic leukemia. It has also been shown that Fludarabine reduces STAT1 phosphorylation and suppress STAT1 protein and transcript levels [30], [31]. On the other hand, the expression of Wnt5a was significantly augmented by pretreatment with STA21 compared to *P. gingivalis* LPS alone. This suggests that STAT3 plays some role in the expression of Wnt5a.

Wnt5a might be up-regulated by *P. gingivalis* LPS and IFN-γ via NF-κB and STAT1 pathways, and induce various cellular functions. Wnt5a activates the non-canonical pathway which results in up-regulation of inflammatory cytokines such as IL-6, IL-8, IL-1β and MIP-1β [11] and these cytokines ultimately induce bone resorption [16], [17]. Moreover, increased levels of Wnt5a and cytokines may affect other mono nuclear cells including T-cells leading sustained inflammation [10] and affect surrounding resident tissue cells, including vascular smooth muscle cells and endothelial cells [32]–[34]. Taken together, Wnt5a may play an important role in periodontal inflammation.

Inflammatory reactions are regulated by cytokines. IFN-γ orchestrates immune response cross-talk with other cytokines or extracellular stimuli such as LPS. For example, pro-inflammatory genes such as inducible nitric-oxide synthase [35], [36] and intercellular adhesion molecule 1 [37] were induced by co-stimulation with IFN-γ and LPS. Here, we also show a synergistic effect of *P. gingivalis* LPS and IFN-γ but not IL-6 or type I IFN (IFN-β) on Wnt5a expression in the human monocytic cell line THP-1. These synergistic effects might be mediated by transcription factors such as NF-κB and STAT1, although additional experiments are needed. Ohmori et al. demonstrated a synergistic effect of NF-κB and STAT1 on iNOS gene induction in Raw264 cells that was activated by LPS or IFN-α, respectively [36]. On the contrary, in our system, we could not observe a synergistic effect of LPS and IFN-α on Wnt5a induction, suggesting that the autocrine loop of LPS-induced IFN-α is not involved in Wnt5a induction. Previously, we reported that NF-κB and STAT3 co-operative role on several genes [38]. In this paper, we used a pharmacological approach to examine STAT3 involvement in Wnt5a induction. Unexpectedly, an inhibitor of STAT3 increased the Wnt5a mRNA level. Furthermore, STAT3 phosphorylation was not observed until 4 hrs after stimulation in THP-1 cells (data not shown). These results suggest that STAT3 does not positively regulate Wnt5a expression, while it is still unclear if STAT3 plays a role as a negative regulator. The relationship between Wnt5a and STAT3 is cell specific [22], [39]. We conclude that Wnt5a gene expression is regulated by NF-κB and STAT1 with different regulatory mechanisms.

## Materials and Methods

### Study population, clinical examination, and sampling of gingival tissue

The Khon Kaen University review committee approved the protocol for human subjects. Twenty-seven subjects were enrolled in this study. All subjects had at least 10 natural teeth and were systemically healthy. After having signed informed consent forms, samples of gingival tissues were obtained from 14 periodontally healthy individuals (control group) and 13 patients with generalized moderate to severe chronic periodontitis [40] during extraction of teeth. Each subject had at least one tooth which was planned for extraction due to severe periodontal destruction and inflammation (chronic periodontitis patient) or for orthodontic treatment or crown lengthening procedure (control subject). The PD, CAL, and bleeding on probing (BOP) of the sampling sites were evaluated using a periodontal probe (Hu-Friedy, Leimen, Germany).

### Human monocyte and human gingival fibroblast

The Tokyo Medical and Dental University review committee approved the protocol for human subjects. After acquiring informed consent, peripheral blood was obtained by venipuncture from 3 healthy volunteers. Peripheral blood mononuclear cells (PBMCs) were isolated by density-gradient centrifugation using Lymphoprep (Axis-Shield, Oslo, Norway). Monocytes were purified by using the MACS Monocyte Isolation Kit II (Miltenyi Biotec, Bergisch Gladbach, Germany). Human gingival fibroblasts (HGF) were isolated from three systemically healthy patients with periodontitis. Three healthy gingival samples were collected during flap surgery. The washed explants were placed in a sterile dish and minced into smaller pieces with a sterile scalpel blade. Attempts were made to remove the epithelium and leave only connective tissue. Fibroblastic cells were allowed to grow out from the explant at 37°C in a humidified atmosphere with 5% CO_2_ in the air until they formed a confluent layer, at which point they were subcultured. Cells subcultured to the fifth passage.

### Cell culture

The human monocytic cell line THP-1 and the human gingival fibroblast cell line HGF-1 were obtained from the American Type Culture Collection (ATCC, Rockville, MD). THP-1 cells and HGF-1 cells were cultured in RPMI 1640 (Lonza, Basel, Switzerland) and DMEM (ATCC) supplemented with 10% heat-inactivated fetal bovine serum (FBS, Hyclone, Logan, UT) and 1% antibiotics (100 U/ml of penicillin and 100 µg/ml of streptomycin). All cells were grown at 37°C in a humid incubator with 5% CO_2_.

### Bacteria, reagents, and antibodies

*Aggregatibacter actinomycetemcomitans* ATCC 43718 and *Porphyromonas gingivalis* 381 were cultured, and sonicated extracts were prepared as described previously [41]. Live *P. gingivalis* 381 (kindly gifted from Dr. Takeuchi), *P. gingivalis* LPS (InvivoGen, San Diego, CA), *E. coli* 055:B5 LPS (Sigma, Taufkirchen, Germany), IL-6, IFN-β, and IFN-γ (R&D systems, Minneapolis, MN) were purchased from the respective companies. Unless otherwise stated, *A. actinomycetemcomitans* and *P. gingivalis* were added to cell cultures at a final concentration of 1 µg/ml. TNF-α was added at a final concentration of 2 ng/ml or 10 ng/ml. IL-6, IFN-β, and IFN-γ were added at a final concentration of 10 ng/ml.

In some experiments, specific signal transduction inhibitors for JAK/STAT (AG490, Calbiochem, San Diego, CA), STAT1 (fludarabine, WAKO, Osaka, Japan), STAT3 (STA21, Santa Cruz Biotechnology, Santa Cruz, CA), NF-κB (MG132, Calbiochem) and IKK (wedelolactone, IKK inhibitor VII, Calbiochem) were added 1 hr prior to stimulation with *P. gingivalis* LPS. When necessary, inhibitors were diluted in DMSO or ethanol. Cell viability was measured by using the Cell Counting Kit-8 (DOJINDO, Kumamoto, Japan). To all unstimulated and control samples, equal amounts of inhibitor solvent were added. Neither DMSO nor ethanol at the concentrations used to prepare inhibitors affected THP-1 cell viability (data not shown).

Antibody for inhibitor κBα (IκBα, sc-371) was purchased from Santa Cruz Biotechnology (Santa Cruz). Anti-β-actin antibody (A5441) was generated by Sigma. HRP-conjugated anti-rabbit IgG (#458) and HRP-conjugated anti-mouse IgG (NA931VS) were purchased from MBL (Nagoya, Japan) and Amersham Pharmacia Bioscience (Buckinghamshire, U.K).

### RNA extraction and RT-PCR

Total RNA from gingival tissues and cells was isolated by using an RNeasy Mini kit (Qiagen) according to the manufacturer's instructions, and concentrations were measured by spectrophotometry. RNA quality was assessed by measuring the ratio of absorbance at 260 nm to the absorbance at 280 nm. Reverse transcription-polymerase chain reaction (RT-PCR) was performed with an RT-PCR kit (One-step RT-PCR kit, Qiagen) using a thermal cycler (GeneAmp® PCR system 2400, Perkin Elmer, Roche, NJ, USA). Specific oligonucleotides were synthesized (Qiagen) based on the published sequences of Wnt5a, forward 5′ TTT TTC TCC TTC GCC CAG GTT GT 3′, reverse 5′ GGC TCA TGG CGT TCA CCA C 3′; β-actin, forward 5′ TGA CGG GGT CAC CCA CAC TGT GCC CAT CTA 3′, reverse, 5′ CTA GAA GCA TTT GCG GTG GAC GAT GGA GGG-3′; and GAPDH forward, 5′ ACC ACA GTC CAT GCC ATC AC 3′, reverse 5′ ACC ACC CTG TTG CTG TA 3′. Real-time RT-PCR was performed with a QuantiTect PrimerAssay and SYBR Green PCR Master Mix (Qiagen) with LightCycler technology (Roche Diagnosis, Mannheim, Germany) and StepOne (Applied Biosystems, Foster City, CA). Wnt5a mRNA expression was displayed as relative expression normalized to β-actin or GAPDH.

### Flow cytometry

Cell surface expression of TLR2 and TLR4 on THP-1 cells was determined by flow cytometry. After washing, cells were stained with FITC anti-mouse/human TLR2 (Abcam, Cambridge, UK) and PE anti-human TLR4 (eBioscience, San Diego, CA). Data were analyzed by the FACS Calibur (Becton Dickinson, San Jose, CA).

### Western blotting

THP-1 cells were lysed with RIPA lysis buffer (1% Nonidet P-40, 0.5% sodium deoxycholate, 0.1% SDS, and protease inhibitor cocktail) for preparation of whole cell extracts. Equivalent amounts of protein (10 µg) were resolved on SDS-PAGE gels, transferred and immobilized on nitrocellulose membranes (Amersham, Little Chalfont, UK), and probed with appropriate primary and secondary antibodies.

### Transfection and luciferase assay

THP-1 cells were transfected with the transfection reagent Lipofectamine™2000 (Invitrogen, Carlsbad, CA) at 3 µl of reagent per one µg of DNA as previously reported [38]. IκB dominant-negative vectors (S32A, S36A double mutant) and pUSE control vectors were obtained from Upstate Biotechnology (Lake Placid, NY). STAT1 vectors were constructed by inserting PCR-amplified STAT1 cDNA fragments into the pcDNA3.1 expression vector (Invitrogen). All plasmids for transfections were prepared by means of the EndoFree Plasmid Maxi Kit (Qiagen). NF-κB luciferase reporter construct [38] and expression vectors were added to 90% confluent cells in 24-well culture plates. At 18 hrs after transfection, cells were stimulated with *P. gingivalis* LPS. After an additional 4 hrs of incubation, the cells were lysed with Passive Lysis Buffer (Promega, Madison, WI). Luciferase activity was measured by using a luminescencer-JNR-II.

### siRNA transfection

RNA interference for STAT1 (Cell Signaling, Danvers, MA), TLR2, TLR4 (Santa Cruz) and negative control siRNA (Cell Signaling) were used. STAT1 siRNA (Cell Signaling, Danvers, MA) and negative control siRNA (Cell Signaling) were used. THP-1 cells were transfected with Lipofectamine™ RNAiMAX Reagent (Invitrogen) according to the manufacturer's protocol and incubated for 18 hrs. A 42% knockdown of STAT1 mRNA was measured. Following transfection, THP-1 cells were stimulated with *P. gingivalis* LPS for 4 hrs.

### Electrophoretic mobility-shift assay (EMSA)

EMSA was performed as described previously [38]. Cells were lysed with NucBuster protein Extraction Kit according to the manufacturer's instructions (Novagen, Darmstadt, Germany). Murine NF-κB oligonucleotide, 5′-AGTTGAGGGGACTTTCCCAGGC-3′, was purchased from Promega. Ten µg of extract was pre-incubated for 20 min at room temperature in 15 µl of buffer (10 mM Tris–HCl pH 7.5, 1 mM EDTA, 1 mM β-mercaptoethanol, 4% glycerol, and 40 mM NaCl) containing 1 µg of poly (dI-dC) and the oligonucleotide labeled with T4 polynucleotide kinase (New England Biolabs, Beverly, MA) and [γ-^32^P]ATP (111 TBq mmol-1, Amersham Pharmacia Biotech, Little Chalfont, UK). Protein–DNA complexes were resolved on 4% TBE polyacrylamide gels and analyzed with a specific antibody.

### Statistical analysis

Data are represented as means ± SD for replicate experiments. The Mann-Whitney U test was used to compare the Wnt5a/β-actin ratios between periodontitis tissue and non-periodontitis tissue. Data were subjected to one-way analysis of variance (ANOVA) using StatView. Fisher's prospected least significance test was used for the post hoc comparison of specific groups. A value of *p*<0.05 was considered statistically significant.
